# Exploring Women’s Perspectives on Receiving AI-Enabled Digital Support for Infant Feeding: Multimethods Cross-Sectional Study

**DOI:** 10.2196/85102

**Published:** 2026-08-05

**Authors:** Sonya Stanley, Jacklyn Jackson, Alison L Brown, Cassandra Lane, Nayerra Hudson, Tessa Delaney, Luke Wolfenden, Rachel Sutherland

**Affiliations:** 1 Hunter New England Local Health District Wallsend, NSW Australia; 2 Population Health Research Program Hunter Medical Research Institute New Lambton, NSW Australia; 3 College of Health, Medicine and Wellbeing University of Newcastle Callaghan, NSW Australia; 4 National Centre of Implementation Science University of Newcastle Callaghan, NSW Australia; 5 Centre for Population Health St Leonards, NSW Australia

**Keywords:** digital health interventions, digital technologies, artificial intelligence, AI in health care, mHealth, infant feeding, mobile health

## Abstract

**Background:**

Infant feeding practices, including breastfeeding, are known to benefit maternal and child health outcomes. Therefore, parent access to evidence-based infant feeding advice is critical. In recent years, there has been increased use of digital health technologies to support infant feeding. Despite its potential, using AI to complement existing health care and connect families to timely infant feeding support remains relatively unexplored.

**Objective:**

This study aims to explore women’s perceptions of using AI-enabled infant feeding support within mobile health (mHealth) interventions. The study investigates (A) openness to AI-enabled support, (B) experiences with existing AI-enabled support, (C) preferences for SMS text messages generated by AI versus “child and family health” nurses, and (D) opinions on infant feeding topics suitable for AI.

**Methods:**

Two data collection activities were undertaken with women (primary caregivers) of infants aged 6-14 months, residing in the Hunter New England Local Health District (HNELHD) of New South Wales, Australia. Different women who received antenatal care in HNELHD were recruited for quantitative and qualitative data collection. Quantitative surveys assessed women’s openness to receiving AI-enabled support (objective A). Descriptive and logistic regression analyses were conducted to explore associations between participant characteristics and openness to AI. Qualitative data collection involved focus groups to explore women’s perceptions and preferences on infant feeding topics suitable for AI (objectives B, C, and D). Thematic analysis was used to analyze focus group transcripts.

**Results:**

A total of 164 women completed the quantitative survey. Approximately 53% (87/164) of participants were open to receiving AI recommendations to see a health professional for infant feeding support, 34% (56/164) were open to AI assessing their breastfeeding experiences, and 41% (67/163) were open to AI providing advice to prevent or address breastfeeding challenges. Fewer Aboriginal and Torres Strait Islander participants were open to receiving AI-generated support (adjusted odds ratio 0.41, 95% CI 0.19-0.92) or advice to see a health professional (adjusted odds ratio 0.29, 95% CI 0.13-0.64). Twelve women participated in 3 online focus groups. Thematic analysis resulted in three overarching themes: (1) opportunities to fill gaps in support, (2) variable confidence engaging with AI for information and advice, and (3) potential convenience of AI and mHealth to offer timely support.

**Conclusions:**

The study highlights the potential of AI and barriers to women’s acceptability and engagement. While women recognized the potential for AI to fill health care gaps in infant feeding support, including after business hours, there was less interest in AI replacing “in-person” support or information easily located via online search. Women’s concerns regarding the credibility and trustworthiness of AI-enabled support should be addressed to maximize their use of emerging AI-enabled tools, embedded within digital technologies and mHealth. There is potential for AI to complement rather than replace usual care.

## Introduction

Digital technologies are expanding as a tool that could transform the delivery of health care and services. AI and mobile devices such as smartphones and tablets offer significant potential for efficient and effective health care delivery [1], particularly in the prevention of noncommunicable diseases and improving mental health [2]. Digital health interventions (DHIs) have been successfully used for population health initiatives delivered at scale [3,4]. Behavior science research also demonstrates that AI has been used in a range of digital behavior change interventions targeting physical activity and nutrition in adults [5]. While AI has the potential to fundamentally change the way health care is delivered, there are challenges to ensure its application in service delivery is appropriate and ethical [6].

Alongside the rapid technological advances, the slower adoption of AI in health care has been linked to ethical considerations, safety, and the perceptions of stakeholders including consumers [7]. A systematic review found positive sentiments toward AI in health care among patients and the public, but concerns related to privacy, familiarity, and trust were also common [8]. Gender differences have also been reported among health care audiences, with less support for AI in health care among women compared to men [9]. These mixed attitudes toward AI in health care suggest that the potential of integrating AI-enabled support within health interventions requires further understanding from the perspective of consumers.

Population health interventions to improve infant feeding are a global policy priority, underpinned by strong evidence of the protective effects for both maternal and child health [10]. Good nutrition including breastfeeding in the first 2000 days can improve child health outcomes and reduce the prevalence of lifestyle-related diseases such as obesity [11]. Breastfeeding also conveys significant maternal health benefits in the immediate and long-term, including healthy weight and reduced risk of ovarian and breast cancers [12]. This is reflected in the Australian Infant Feeding Guidelines, which recommend exclusive breastfeeding until around 6 months of age [13]. Despite these evidence-based guidelines, optimal infant feeding practices remain challenging for women [14].

Women are more likely to breastfeed when they receive adequate in-hospital support and partners are involved in education [15]. In Australia, there are existing services that support women in hospitals and community-based settings such as child and family health (CFH) services. Beyond initial hospital care, community support for women continues to be highly influential on breastfeeding [16].

Around 90% of Australian women intend to and start breastfeeding in the hospital [17]. Despite their intentions, breastfeeding rates decline by around one-quarter after leaving the hospital (27%) [17]. If support is delayed or discontinued between the hospital and returning home, it can significantly impact breastfeeding, highlighting a potential service gap between settings [18]. There is an opportunity for innovative service delivery to engage families with timely, evidence-based infant feeding support.

DHIs have already been trialed as part of population health initiatives to support infant feeding. Common examples accessible via mobile devices (mobile health [mHealth]) include apps [19,20], SMS text messages [21], and/or telehealth with health professionals [22] or volunteers [23]. Using digital health technologies for infant feeding support has been shown to be acceptable to mothers during the first 2000 days [24]. While parents have reported that mHealth support is accessible and convenient for health information [20], concerns regarding the trust and credibility of information have also been highlighted [25].

Despite the growing role of digital health and high connectivity to technology among Australians, there is limited evidence on the use of AI-enabled support for infant feeding and women’s perceptions of using digital mHealth. This study used quantitative surveys and qualitative focus groups with women who are the primary caregivers of infants aged 6 to 14 months. Their insights into how AI could support infant feeding and the potential enablers and barriers to engaging with AI-enabled support are analyzed in this paper.

The aim of this study is to inform the potential application of AI in mHealth to complement infant feeding support, from the perspective of consumers. The study investigated women’s perceptions of AI-enabled infant feeding support offered as part of a DHI. There are 4 specific objectives to explore women’s (A) openness to receiving AI-enabled infant feeding support, (B) experiences with existing forms of AI-enabled infant feeding support, (C) preferences for SMS text messages generated by AI versus CFH nurses for evidence-based infant feeding advice, and (D) opinions on infant feeding topics that could be suitable for AI-enabled support.

## Methods

### Study Design

To inform the 4 research objectives, we used a multimethods research design using quantitative and qualitative methods without data integration [26]. Our study was designed to elicit a broad range of perspectives on the complex issue of infant feeding.

### Study Population

Participants included women with an infant 6-14 months residing in the Hunter New England Local Health District (HNELHD) of New South Wales, Australia. The HNELHD is a socioeconomically and geographically diverse region encompassing major metropolitan and regional/remote locations [27]. There are approximately 10,000 births annually in the district, accounting for just over 10% of births in New South Wales, Australia [28].

Electronic medical records and antenatal survey data were used to identify the sampling frame. Convenience sampling methods were used in that women were eligible if they had previously agreed to be contacted to participate in research related to their postpartum care, from public maternity services. Women with infants around the same age (6-14 months) were recruited to ensure participants had recent experience with infant feeding. Women who met the eligibility criteria were mailed an information letter with the details of the survey or focus group. Those who did not reply to the letter received a follow-up call from a female researcher. Different women were recruited for each data collection to avoid any overlap in responses. Local cultural consultation informed the recruitment of Aboriginal and Torres Strait Islander women. Recruitment and survey data were captured and stored via REDCap [29].

### Quantitative Survey

Women who were 12-14 months postpartum were eligible to participate in the survey, conducted by computer-assisted telephone interviewing (CATI). Women who did not identify as Aboriginal and/or Torres Strait Islander received up to 10 phone attempts over a 2-week period to invite participation. Verbal consent to participate in the survey was sought from women at the time of the interview call. Women who declined to participate in the CATI were offered the option to complete the survey online. Prior to accessing the online survey, women were reminded that participation was voluntary and that the survey could be declined at any point.

Women identifying as Aboriginal and/or Torres Strait Islander were sent an SMS text message after the mail-out of the information statement, offering the option to complete the survey via CATI, online survey, or decline participation. Those who opted to complete the survey online were sent an individual survey link to their mobile number that was active for 2 weeks.

### Qualitative Focus Groups

Women were eligible for the focus groups if they were 6-12 months postpartum and had access to an internet-enabled device with a camera (eg, mobile phone or computer) to facilitate online participation. An online screening and eligibility tool accessible via a weblink or QR code was offered to focus group participants, and it also enabled participants to nominate their preferred availability.

Women who identified as Aboriginal and/or Torres Strait Islander were offered the opportunity to complete the screening and eligibility tool with an Aboriginal and/or Torres Strait Islander staff member. They were also asked if they preferred to participate in a focus group with other Aboriginal and/or Torres Strait Islander women.

A confirmation email for the focus group and a secure, password-protected weblink were sent to registered participants. A reminder SMS text message was sent to participants on the business day prior to the scheduled focus group.

### Data Collection

#### Quantitative Survey

Quantitative data were obtained via surveys conducted to assess women’s openness to receiving AI-enabled support for breastfeeding (objective A). Quantitative cross-sectional surveys were conducted via CATI. The survey was conducted between August 2023 and July 2024. Women answered demographic questions including Aboriginal and/or Torres Strait Islander origin, residing postcode, current employment status, and child’s date of birth. It was developed and stored using REDCap [29].

The survey was designed to evaluate the impact of an SMS text messaging intervention for new families in the first 2000 days [30,31]. The survey items include validated and adapted tools to assess key outcome measures including engagement, acceptability, and potential effectiveness.

The survey was reviewed by key partners (CFH nurses, dietitians, and Aboriginal health care workers) and pilot-tested prior to use. The survey items related to AI were only asked to a subset of respondents, including:

1. Those who had indicated previous experience breastfeeding, and
2. Those who had not received the locally provided SMS text messaging that included content to promote breastfeeding.

Prior to participants responding to the AI survey items, they were given the following preamble:

In the future, some health care services may use computer programs that have the ability to think or act in ways that are similar to how humans think or act. This may allow them to be used to help in providing information and support to people in different areas of their health care. We want to understand your views on this type of digital service and technology if it were available to provide you with support in your breastfeeding journey.

This was followed by three survey items to explore women’s openness to AI-generated infant feeding support (objective A):

1. How open are you to allow a digital service, as described to assess your breastfeeding experience?
2. How open are you to allow a digital service, as described to give advice on how to prevent or address breastfeeding issues and challenges?
3. How open are you to allow a digital service, as described to recommend when to see a health professional about your breastfeeding experiences?

Responses were recorded using a 5-point Likert scale (not open, somewhat open, open, very open, and extremely open), a method that is consistent with other validated measures used to assess openness to AI-driven health care technologies [32].

#### Qualitative Focus Groups

Focus groups were designed to understand the complexity of infant feeding and women’s experiences, preferences, and opinions related to AI-enabled support (objectives B, C, and D). The qualitative component was intended to gain deeper insights from participants by providing a supportive group setting for sharing, reflection, and connection. The focus groups were conducted online for up to 45 minutes. Online Zoom videoconferencing was used to minimize any travel burden and restrictions based on participants’ geographic location. An experienced facilitator led the discussion, and an additional member from the research team acted as an observer. The design and reporting of the focus groups were guided by the COREQ (Consolidated Criteria for Reporting Qualitative Research) checklist [33].

The focus groups were conducted using a semistructured guide, developed by the research team. The guide was designed to explore the research objectives using open-ended questions and prompts for participants to openly share their perceptions of AI-generated content. The guide covered three topic areas, aligned with the research objectives: (1) experiences with AI-enabled infant feeding support (objective B), (2) perceptions of content generated for SMS text messages by AI versus CFH nurses (objective C), and (3) opinions on infant feeding topics that could be addressed via AI-enabled support (objective D).

At the start of each focus group, the facilitator sought verbal consent from participants for their responses to be audio-recorded. Participants were reminded of the study objectives and group expectations for confidentiality.

The semistructured methodology provided participants with the opportunity to lead aspects of the conversation and discuss their experiences related to infant feeding. The facilitator tailored the pace and progression of open-ended questions and used group facilitation strategies to ensure all participants had an opportunity to comment on each topic. During each focus group, participants were also shown SMS text messages without revealing how they were generated (with nurses versus AI, used by the research team). Participants were first invited to share their perceptions; afterward, they were told how each message was developed and then asked their opinion on the source.

All focus groups were audio-recorded and transcribed postinterview. The transcripts were deidentified and checked for accuracy by comparison with the recordings.

### Data Analysis

#### Quantitative Survey

Data were analyzed using the SAS (version 9.3; SAS Institute) statistical software package. Descriptive statistics were used to describe participant characteristics and openness to receiving AI-enabled breastfeeding support. Women reporting on their attitudes to receiving AI-enabled breastfeeding support were dichotomized into “open” (open, very open, and extremely open) and “not open” (somewhat open and not open). Associations between participant characteristics and openness to AI were explored using logistic regression models. Characteristics included age, socioeconomic status, geographical remoteness, employment status, level of education, number of pregnancies, and Indigenous status. For each outcome, both unadjusted models (one per characteristic) and a single multivariable-adjusted model (including all 7 characteristics) were conducted.

#### Qualitative Focus Groups

Qualitative transcripts were analyzed using reflexive thematic analysis [34]. The audio recordings, transcriptions, and observation notes were reviewed by the research team as part of the data familiarization process. Data from the transcripts were managed and coded using NVivo 14 (Lumivero) statistical software.

The analysis was structured to align with the 3 topics of experiences, perceptions, and opinions of AI-enabled support. Two members of the research team coded the transcripts independently, identifying relevant quotes and emerging themes in the data that reflected the research topics. The analysts shared and discussed their initial coding and collaborated to organize the codes into themes using a reflexive, open, and critical approach. Throughout this process, the research team reflected on their perspectives and experiences, considering how this shaped the development of codes and themes.

The feedback on the example SMS text messages was also analyzed for the percentage of correct identification of messages generated by AI versus CFH nurses.

### Ethical Considerations

Ethical approval to undertake the quantitative survey was obtained from the Hunter New England Human Research Ethics Committee (2019/ETH00998), Aboriginal Health and Medical Research Council (1236/16), and the University of Newcastle (H-2017-0032). Ethical approval for the qualitative study was obtained from the Hunter New England Human Research Ethics Committee (2020/ETH02612), the University of Newcastle (H-2020-0086), and the Aboriginal Health and Medical Research Council (1727/20). All specific cultural safety protocols were followed for this study.

This research was conducted in line with informed consent guidelines and regulations regarding the protection of personal information, privacy, and human rights. Data were deidentified and securely stored according to all ethical approvals. Participants in the focus groups received a gift voucher of Aus $50 (Aus $1=US $0.67 as of December 2023) to compensate for their time.

### Reflexivity Statement

The research team consisted of health professionals with expertise in nutrition and public health. Members of the team were qualified dietitians (ALB, TD, JJ, NH, RS, and SS) and had formal research qualifications (ALB, TD, JJ, CL, RS, and LW). Several team members also had personal experience with breastfeeding (ALB, TD, JJ, NH, and RS) at the time of the study. All team members had experience in health promotion and using population-based approaches to support families in the first 2000 days. The focus group facilitators were women and health professionals with experience in group facilitation and health research. The facilitators were also involved in the development of the interview guide. The research team shares a common understanding that our perspectives and experiences informed and enhanced the design and analysis of the qualitative study.

## Results

### Quantitative Survey Results

Of the 626 women approached, 283 eligible participants completed the survey (45% response rate). Of these 283 participants, 104 participants had received the locally provided text-message program, and a further 15 participants indicated that they never attempted to breastfeed their child, leaving 164 women who completed the survey items related to AI-generated breastfeeding support. The mean age of participants was 31.1 (SD 5.4) years, and most (115/163, 71%) had obtained a tertiary level of education. Most participants (104/164, 63%) were employed, and 67% (108/162) resided in an area of most socioeconomic disadvantage (Table 1).

Women’s openness to receiving AI-generated breastfeeding support was moderate to low (Table 2). Approximately half (87/164, 53%) reported being open to AI recommending when they should see a health professional about their breastfeeding experience. Fewer were open to engaging with AI for advice on how to prevent or address breastfeeding challenges (67/164, 41%) or assess their breastfeeding experience (56/164, 34%). Women’s openness to receiving AI-generated breastfeeding support was significantly associated with participant Aboriginal and/or Torres Strait Islander status. Significantly fewer Aboriginal and Torres Strait Islander participants were open to receiving advice on how to prevent or address breastfeeding issues and challenges (adjusted odds ratio [OR] 0.41, 95% CI 0.19-0.92; *P*=.03), or having AI recommend when to see a health professional about their breastfeeding experience (adjusted OR 0.29, 95% CI 0.13-0.64; *P*=.002). No other participant demographics significantly impacted participant openness to AI-generated breastfeeding support (Multimedia Appendices 1-3).

**Table 1:** Quantitative study participant characteristics (n=164).

Participant characteristics			Values, n (%)^a^
Age (in years; n=145), mean (SD)			31.1 (5.4)
**Age of baby in months (n=145)**			
	12 months	100 (69)	
	13 months	35 (24)	
	14 months	10 (7)	
Identifies as Aboriginal and/or Torres Strait Islander (n=164)			50 (31)
**Highest level of education (n=163)**			
	High school or less	48 (29)	
	Tertiary	115 (71)	
**Employment status (n=164)**			
	Employed	104 (63)	
	Maternity leave (paid or unpaid)	14 (9)	
	Unemployed	46 (28)	
**Socioeconomic status^b^ (n=162)**			
	Most disadvantaged	108 (67)	
	Least disadvantaged	54 (33)	
**Level of remoteness^c^ (n=162)**			
	Major cities	95 (59)	
	Regional/remote	67 (41)	

^a^Values are the number of participants and proportions [n (%)] unless otherwise stated.

^b^Based on socioeconomic indexes for areas, using residential postcode [35].

^c^Based on the access/remoteness index of Australia, using residential postcode [36].

**Table 2:** Openness to receiving AI-generated breastfeeding support (n=164).

Potential for AI-generated support	Proportion of women open to AI^a^, n (%)
Assess women’s breastfeeding experience	56 (34)
Give advice on how to prevent or address breastfeeding issues and challenges	67 (41)
Recommend when to see a health professional about women’s breastfeeding experience	87 (53)

^a^ “Openness” combined “open,” “very open,” and “extremely open” responses.

### Qualitative Focus Group Results

A total of 140 women were invited to participate in online focus groups, 20 women registered, and 12 women participated in a focus group; 10 (83%) of whom were first-time parents. All participants identified as female, had a child aged 6-12 months, and had experience breastfeeding. None of the participants identified as having Aboriginal or Torres Strait Islander origins. There were 64% (7/11) of participants who were employed and 67% (8/12) living in a metropolitan city (Table 3).

**Table 3:** Quantitative study participant characteristics (n=12).

Participant characteristics			Values, n (%)
Identifies as Aboriginal and/or Torres Strait Islander (n=12)			0 (0)
**Employment status (n=11)**			
	Employed (part-time or casual)	7 (64)	
	Maternity leave (paid or unpaid)	2 (18)	
	Unemployed	2 (18)	
**Level of remoteness^a^ (n=12)**			
	Major cities (metropolitan)	8 (67)	
	Regional/remote (small/medium rural)	4 (33)	

^a^Based on the modified Monash model.

**Figure 1:**
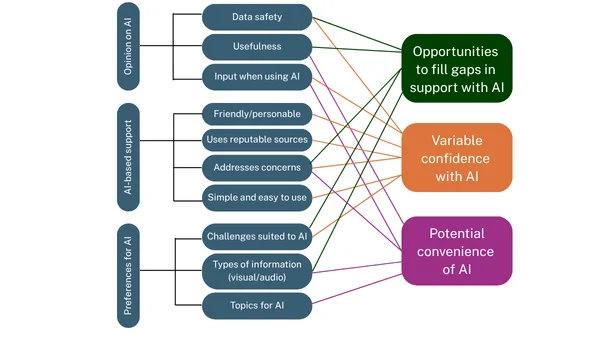
Focus group results: a framework of the key factors influencing AI and infant feeding support.

The analysis led to three overarching themes related to the qualitative research objectives: (1) opportunities to fill gaps in infant feeding support, (2) variable confidence engaging with AI for information and advice on infant feeding, and (3) potential convenience of AI and mHealth to offer timely infant feeding support (Figure 1).

### Theme 1: Opportunities to Fill Gaps in Support

Participants expressed the opinion that AI-enabled support could have advantages that complement existing health care services and fill common gaps in support. While many participants spoke about the usefulness of searching online for infant feeding support, the limitations of AI were also discussed and there was a shared sentiment to suggest AI was commonly reserved for “stuff you can’t find” (via Google).

You've already done that really in-depth Google search or you've already done what you can find and it's the stuff that you can't find is the stuff that you need help with...if that's information that's already available in the websites or the Internet, it's probably not that useful.

Participant 1, metro

Sometimes I feel like Google can be overwhelming and you don't know where the information's coming from.

Participant 2, metro

...And then you have to call and then sometimes calling's just too overwhelming when you're in that frantic state of needing help.

Participant 3, metro

Participants shared their views on the potential advantages for a greater use of AI-enabled support. This centered around timely, 24/7 advice to address infant feeding challenges at different stages of child development.

I find that it's just there's been a lot of issues that have come up past that 6 weeks and things change all the time. I guess, some more support later down the track.

Participant 4, metro

I think the 24/7 availabilities are always a good thing. It's very reassuring that you could do it obviously anytime day or night.

Participant 3, metro

A wide range of general infant feeding topics were suggested to be appropriate for AI-enabled support. These related to supply, latching technique, baby’s weight, and transitional stages of infant feeding. Participants also shared their opinions on key challenges including managing expectations on “normal” and improving gaps in communication and routine care from health professionals:

And I think that was the biggest thing...just knowing it's normal, it's going to get better because at that stage I was like, how long is this going to last for?

Participant 5, rural

Any sort of support for that first few months and expectations of the changes that happen that you are going to have so many...because you go into a panic, you don’t know.

Participant 6, metro

### Theme 2: Variable Confidence

Participants spoke about their confidence in AI in relation to data safety, reputable sources of information, and the ability to address their concerns and challenges with infant feeding. Overall, the feedback was generally positive when discussing the level of confidence engaging with AI, noting that accurate and reputable sources of information were important:

I think it's fine as long as whoever's programming is using reputable, accurate sources to generate the information. I'm all for it.

Participant 7, metro

I think as long as the information is correct, I have no issue with it being AI versus an actual person.

Participant 8, rural

Several participants raised concerns in relation to online data safety and highlighted the importance of reputable and accurate information and sharing/accessing visual resources when engaging with AI. While no one completely dismissed AI, one participant commented on a lack of confidence in their ability to describe a problem:

I'm like just Facebook and data mining and what have you. That just wigs me out a little bit. I would probably want it to be in its own specific, more safe data thing especially if you send back a picture of you feeding your child.

Participant 9, metro

I would find it hard to phrase my question concisely...it's hard to be really describing the problem in words...

Participant 10, rural

The importance of access to a “real person” was highlighted by several participants, including for those living in rural areas, with some caveats. This related to specific expectations that person-based advice should be “more than just a website,” beyond the information that participants could find via their own online search. Participants also expressed a lack of confidence when given inconsistent advice from different health professionals.

...I did find it really helpful talking to a person because I can't even remember the question that I asked, but it was very detailed and specific to me.

Participant 3, metro

It's always good to be able to talk to a real person. But if they are just directing you a lot to the website, I agree that for some people that might not be that helpful...

Participant 9, metro

...sometimes you feel so isolated...It's just nice to know that there's someone there that can help you straight away sort of thing. Especially if you live remotely.

Participant 11, rural

### Theme 3: Potential Convenience

Participants expressed that AI-generated support could offer convenient infant feeding support. First, it could address concerns in a more timely manner compared to other options. For example, several participants reported the use of telephone-based support that was not always convenient as a parent with an infant, particularly after business hours. As such, the late-night hours were suggested as a window of opportunity for AI-enabled support, when other support options may not be available.

...a phone call sometimes (is) not the easiest thing to do when you've got a screaming baby and you are upset or whatever. You don't really want to sit there on the phone...

Participant 3, metro

I think having something that you can access at any point of the day, especially if you're sleep deprived, your baby's screaming at 3AM and you can't figure out what's going wrong. I think that's where AI really comes in handy.

Participant 9, metro

And I remember googling all hours of the night because I was in that panic stage of, is this right? Is this right?

Participant 6, metro

Participants also suggested that AI-enabled support could provide access to visual/audio support. Many participants reported they had sought digital content such as online images and videos for additional guidance. Visual and audio resources were discussed as helpful features to navigate infant feeding challenges, rather than only reading online information:

I would've really loved more videos, sound on because some videos actually have background music, just accompanied with instrumental music.

Participant 10, rural

...but being a first-time mum, first baby visual stuff was very helpful to me.

Participant 3, rural

Participants highlighted user input in relation to the convenience of AI, revealing contrasting perspectives. In some instances, AI was suggested as a convenient means for refining questions, while others felt that the input requirements of AI could be demanding.

And I guess if you can have that backwards and forwards to say, oh, that's not exactly what I was looking for. It was more of this. And then if the AI can respond with a different response, that might be a little bit more helpful.

Participant 1, metro

And that's where an actual person to speak with is also probably my number 1 pick because you can talk to them about your specific situations.

Participant 6, metro

Several participants also commented on the convenience and simplicity of being able to ask questions and receive pictorial and targeted information.

...the AI-generated chat bot, I definitely would ask that a question and I would've found those pictures helpful. That's probably what I would've been looking for in the first few days...

Participant 5, metro

### Feedback on the Identification of Text Messages Generated by AI vs CFH Nurses

Most focus group participants (9/12, 75%) did not correctly identify which message was generated by AI and which was written with expert CFH nurses. Participants indicated that the AI message seemed more friendly while appearing to be a reputable source of information. Moreover, participants were generally unconcerned whether a message contained AI or CFH nurse-developed content.

I guess I wouldn't mind if it was a (CFH) nurse or AI (chat)bot. Because I'm assuming the AI is being fed information that nurses would give...

Participant 9, metro

It wouldn't bother me if it's AI-generated. It wouldn't be noticeable whether it was or not to me. I'd be happy with either...

Participant 5, rural

## Discussion

### Principal Findings

This study investigated the perceptions of mothers regarding AI-enabled infant feeding support using multimethods. The quantitative survey data showed that mothers are open to AI only for limited aspects of infant feeding. The qualitative results from the focus groups highlighted that gaps in existing care, confidence in AI, and convenience, including timing, are also highly influential.

### Comparison With Prior Work

Our research found that mothers were less open to receiving AI-enabled support with increasing AI appraisal of their breastfeeding experience. These findings align with existing literature indicating that while consumers are generally comfortable engaging with AI for administrative tasks like scheduling appointments, using AI to support clinical decisions and patient monitoring is less supported [37,38]. Consistent with the literature exploring adoption of AI [39], the qualitative findings suggest that mothers are open to using AI to assist with “filling the gaps” in support. Despite being time-poor and sharing concerns about health care gaps, mothers were less interested in engaging with AI-enabled support that would replace the care provided by health professionals.

This study also found participant openness to AI was significantly lower in Aboriginal and Torres Strait Islander mothers, suggesting a potential cultural barrier for AI-assisted infant feeding support. Previous research has found specific concerns among Indigenous peoples for AI, such as concerns over data sharing, non-Indigenous biases, and limited Indigenous participation in AI [40]. This result is based on the quantitative survey as there were no Aboriginal women who participated in the focus groups, despite recruitment strategies. More research is needed to explore whether there are potential cultural barriers to engaging with AI-enabled infant feeding support.

The focus group participants were more open to receiving AI-enabled support outside usual business hours or when routine health care is less frequent. As internet access has expanded, so have the behaviors of online help-seeking to find health information that supplements existing health care. Among new parents, this is often regarding infant feeding challenges and developmental milestones [41]. Mothers tend to favor online platforms to acquire information due to their anonymity and fear of judgment using other methods [41]. This represents an opportunity for women to use AI to access the “stuff you can’t find” on infant feeding and to improve the quality and specificity of the information returned, especially during periods of “panic.”

Women’s confidence in AI-enabled support may influence their openness and trust with its content. In this study, focus group participants reported that “reputable,” “legitimate,” and “accurate sources to generate the information” were key to their confidence and trust to engage with AI-enabled support. A systematic review exploring AI in health care found that consumers’ lack of trust in AI was primarily related to data privacy, patient safety, technology, and fears of full automation [7]. Furthermore, a survey (n=307) of US consumers found that technological, ethical (trust factors), and regulatory concerns significantly contributed to the perceived risk of using AI in health care [42].

Our preliminary findings that there was a lack of differentiation when AI was used to generate messages and safety concerns regarding AI are both likely drivers for consumer preferences on how AI is used in health care. This suggests there is potential application of AI into processes such as developing or refining health-related SMS text messages, while maintaining clinician oversight to retain trust. Our study also reinforces a complementary approach for AI alongside in-person support, rather than replacement. It will be important to consider how to systematically address these barriers to integrating AI into preventive health care in the first 2000 days.

Participants in this study were positive about the convenience of AI-enabled support, compared to other forms of support, including telephone hotlines or online searches. Mothers also indicated that AI could offer a more convenient option for accessing timely and targeted information, particularly after hours. This is consistent with previous research that identified the availability and ease of use as potential advantages of AI [7]. Together with our findings, this suggests that easily accessible and relevant information is likely to be important for AI-enabled infant feeding support.

### Strengths and Limitations

This study has several strengths and limitations. The sampling approach used may have limited the representativeness of the overall findings. Future Australian-based research should aim to recruit more representative samples from priority populations for focus groups, including Aboriginal and Torres Strait Islander people. It is possible that social desirability bias may have influenced participants to change their responses based on the wider focus group. However, several factors strengthen the credibility of the findings. The research team meaningfully engaged with focus group participants and intentionally used strategies to create a safe, welcoming space for women to discuss their experiences. Participants in the focus groups shared diverse perspectives and personal stories, indicating a willingness to share openly, providing trustworthy data. Moreover, the findings complement existing literature on common reservations about using AI [37]. Participants were also sampled from a diverse region including metropolitan and rural locations, enhancing the transferability of our findings.

Finally, using two data collection methods enabled the researchers to analyze responses from a larger and more diverse sample compared to using one method alone, and provided rich, reliable data to address 4 distinct and complementary objectives. However, the datasets were not integrated through methodological triangulation, which would have provided a more intricate exploration of women’s perspectives [43]. Given the modest qualitative dataset and the feasibility constraints for the timing of this research, a multimethods design without integration was deemed most suitable. Future studies could include prospective planning to support integrated multimethods research to further explore women’s perspectives of AI support.

### Conclusions

DHIs have already shown promise as a means of improving population health. This study adds new findings to the potential application of AI in mHealth to complement infant feeding support, from the perspective of consumers. Our findings indicate that their openness to AI is based on a strong preference for AI integration rather than replacement with existing clinical care. Our findings also underline the importance of using evidence-based information, oversight from trusted health practitioners, and transparency of AI use, as part of ethical practice. Given the rapid changes in AI technology, this study could be repeated to monitor consumer preferences. Overall, there are nuanced perceptions of engaging parents with AI-enabled infant feeding support and common concerns are consistently reported. AI has significant potential for integration into preventive health care interventions, but consumer perspectives should also be considered in the application of emerging AI technologies.

## Appendix

Multimedia Appendix 1 Association between participant demographics and openness to AI assessing women’s breastfeeding experience.

Multimedia Appendix 2 Association between participant demographics and openness to AI giving advice on how to prevent or address breastfeeding issues and challenges.

Multimedia Appendix 3 Association between participant demographics and openness to AI recommending when to see a health professional about their breastfeeding experience.

## Abbreviations

CATI: computer-assisted telephone interviewing
CFH: child and family health
COREQ: Consolidated Criteria for Reporting Qualitative Research
DHI: digital health intervention
HNELHD: Hunter New England Local Health District
mHealth: mobile health
OR: odds ratio

## Data Availability

The study materials (including the interview guide) and datasets generated during this study are not publicly available due to the sensitive nature of the topic but may be available from the corresponding author on reasonable request and subject to ethical standards.
