# Supplementary material for: Exploring Women’s Perspectives on Receiving AI-Enabled Digital Support for Infant Feeding: Multimethods Cross-Sectional Study
Source: JMIR Form Res. 2026 Aug 5;10:e85102. doi: 10.2196/85102 (PMC13490937; doi:10.2196/85102)
Supplement: Supplementary file 1 [file formative_v10i1e85102_app1.docx]

Association between participant demographics and openness to artificial intelligence assessing women’s breastfeeding experience

| Participant demographic | Open  N (%) | Crude Analysis | | Adjusted Analysis | |
| --- | --- | --- | --- | --- | --- |
|  |  | Odds Ratio (95% CI) | P-value | Odds Ratio (95% CI) | P-value |
| **Socioeconomic Status** | | | | | |
| Most disadvantaged | 35 (32%) | 0.75  (0.38-1.49) | 0.41 | 0.70  (0.31-1.58) | 0.39 |
| Least disadvantaged | 21 (39%) | 1 (reference) |  | 1 (reference) |  |
| **Level of remoteness** | | | | | |
| Major city | 34 (36%) | 1.14 (0.59-2.21) | 0.70 | 0.78 (0.35-1.75) | 0.55 |
| Inner/outer regional or remote | 22 (33%) | 1 (reference) |  | 1 (reference) |  |
| **Employment Status** | | | | | |
| Employed | 36 (35%) | 0.99 (0.48-2.06) | 0.90 | 0.78 (0.33-1.82) | 0.81 |
| Maternity leave | 4 (29%) | 0.75 (0.20-2.78) |  | 0.69 (0.17-2.78) |  |
| Unemployed | 16 (35%) | 1 (reference) |  | 1 (reference) |  |
| **First pregnancy** | | | | | |
| Yes | 19 (40%) | 1.47 (0.73-2.96) | 0.28 | 1.40 (0.64-3.05) | 0.40 |
| No | 37 (32%) | 1 (reference) |  | 1 (reference) |  |
| **Level of education** | | | | | |
| High school or less | 16 (33%) | 0.97 (0.48-1.99) | 0.94 | 0.89 (0.38-2.08) | 0.79 |
| Tertiary | 39 (34%) | 1 (reference) |  | 1 (reference) |  |
| **Mother’s Age** |  | 0.97 (0.92-1.03) | 0.38 | 0.98  (0.91-1.06) | 0.59 |
| **Identifies as Aboriginal and/or Torres Strait Islander** | | | | | |
| Yes | 13 (26%) | 0.58 (0.28 – 1.21) | 0.15 | 0.57 (0.25- 1.29) | 0.18 |
| No | 43 (38%) | 1 (reference) |  | 1 (reference) |  |
