# Supplementary material for: Exploring Women’s Perspectives on Receiving AI-Enabled Digital Support for Infant Feeding: Multimethods Cross-Sectional Study
Source: JMIR Form Res. 2026 Aug 5;10:e85102. doi: 10.2196/85102 (PMC13490937; doi:10.2196/85102)
Supplement: Supplementary file 2 [file formative_v10i1e85102_app2.docx]

Association between participant demographics and openness to artificial intelligence giving advice on how to prevent or address breastfeeding issues and challenges

| Participant demographic | Open  N (%) | Crude Analysis | | Adjusted Analysis | |
| --- | --- | --- | --- | --- | --- |
|  |  | Odds Ratio (95% CI) | P-value | Odds Ratio (95% CI) | P-value |
| **Socioeconomic Status** | | | | | |
| Most disadvantaged | 44 (41%) | 97 (0.50-1.89) | 0.93 | 0.93 (0.42-2.07) | 0.86 |
| Least disadvantaged | 22 (42%) | 1 (reference) |  | 1 (reference) |  |
| **Level of remoteness** | | | | | |
| Major city | 38 (40%) | 0.95 (0.50-1.79) | 0.86 | 0.72 (0.33-1.56) | 0.40 |
| Inner/outer regional or remote | 28 (42%) | 1 (reference) |  | 1 (reference) |  |
| **Employment Status** | | | | | |
| Employed | 42 (41%) | 0.98 (0.48-1.98) | 0.99 | 0.86 (0.38-1.97) | 0.80 |
| Maternity leave | 6 (43%) | 1.07 (0.32-3.58) |  | 1.28 (0.34-4.76) |  |
| Unemployed | 19 (41%) | 1 (reference) |  | 1 (reference) |  |
| **First pregnancy** | | | | | |
| Yes | 20 (43%) | 1.09 (0.55-2.16) | 0.81 | 0.92 (0.42-1.99) | 0.83 |
| No | 47 (41%) | 1 (reference) |  | 1 (reference) |  |
| **Level of education** | | | | | |
| High school or less | 21 (44%) | 1.19 (0.60-2.36) | 0.61 | 1.23 (0.54-2.78) | 0.63 |
| Tertiary | 45 (39%) | 1 (reference) |  | 1 (reference) |  |
| **Mother’s Age** |  | 0.98 (0.92-1.04) | 0.48 | 0.98 (0.92-1.06) | 0.68 |
| **Identifies as Aboriginal and/or Torres Strait Islander** | | | | | |
| Yes | 15 (30%) | 0.50 (0.25-1.02) | 0.057 | 0.41(0.19 – 0.92) | 0.03 |
| No | 52 (46%) | 1 (reference) |  | 1 (reference) |  |
