# Supplementary material for: Exploring Women’s Perspectives on Receiving AI-Enabled Digital Support for Infant Feeding: Multimethods Cross-Sectional Study
Source: JMIR Form Res. 2026 Aug 5;10:e85102. doi: 10.2196/85102 (PMC13490937; doi:10.2196/85102)
Supplement: Supplementary file 3 [file formative_v10i1e85102_app3.docx]

Association between participant demographics and openness to artificial intelligence recommending when to see a health professional about their breastfeeding experience

| Participant demographic | Open  N (%) | Crude Analysis | | Adjusted Analysis | |
| --- | --- | --- | --- | --- | --- |
|  |  | Odds Ratio (95% CI) | P-value | Odds Ratio (95% CI) | P-value |
| **Socioeconomic Status** | | | | | |
| Most disadvantaged | 55 (51%) | 0.77 (0.40-1.49) | 0.44 | 1.18 (0.53-2.62) | 0.69 |
| Least disadvantaged | 31 (57%) | 1 (reference) |  | 1 (reference) |  |
| **Level of remoteness** | | | | | |
| Major city | 56 (59%) | 1.77 (0.94-3.33) | 0.08 | 1.52 (0.70-3.31) | 0.29 |
| Inner/outer regional or remote | 30 (45%) | 1 (reference) |  | 1 (reference) |  |
| **Employment Status** | | | | | |
| Employed | 56 (54%) | 1.17 (0.58-2.34) | 0.86 | 0.94 (0.41-2.17) | 0.66 |
| Maternity leave | 8 (57%) | 1.33 (0.40-4.45) |  | 1.69 (0.42-6.76) |  |
| Unemployed | 23 (50%) | 1 (reference) |  | 1 (reference) |  |
| **First pregnancy** | | | | | |
| Yes | 25 (53%) | 1.01 (0.51-1.99) | 0.98 | 0.81 (0.37-1.75) | 0.59 |
| No | 62 (53%) | 1 (reference) |  | 1 (reference) |  |
| **Level of education** | | | | | |
| High school or less | 27 (56%) | 1.22 (0.62-2.40) | 0.56 | 1.79 (0.77-4.20) | 0.18 |
| Tertiary | 59 (51%) | 1 (reference) |  | 1 (reference) |  |
| **Mother’s Age** |  | 1.00 (0.94-1.05) | 0.87 | 1.02 (0.95-1.09) | 0.66 |
| **Identifies as Aboriginal and/or Torres Strait Islander** | | | | | |
| Yes | 17 (34%) | 0.32 (0.16-0.65) | 0.001 | 0.29 (0.13- 0.64) | 0.002 |
| No | 70 (61%) | 1 (reference) |  | 1 (reference) |  |
